# Inventory of real world data sources in Parkinson’s disease

**DOI:** 10.1186/s12883-017-0985-0

**Published:** 2017-12-08

**Authors:** Audrey Tanguy, Linus Jönsson, Lianna Ishihara

**Affiliations:** Lundbeck SAS, 37-45 Quai du Président Roosevelt, CEDEX 92445 Issy-les-Moulineaux, France

**Keywords:** Parkinson disease, Rating scales, Longitudinal, Cohort studies, Real-world

## Abstract

**Background:**

Real world data have an important role to play in the evaluation of epidemiology and burden of disease; and in assisting health-care decision-makers, especially related to coverage and payment decisions. However, there is currently no overview of the existing longitudinal real world data sources in Parkinson’s disease (PD) in the USA. Such an assessment can be very helpful, to support a future effort to harmonize real world data collection and use the available resources in an optimal way.

**Methods:**

The objective of this comprehensive literature review is to systematically identify and describe the longitudinal, real world data sources in PD in the USA, and to provide a summary of their measurements (categorized into 8 main dimensions: motor and neurological functions, cognition, psychiatry, activities of daily living, sleep, quality of life, autonomic symptoms and other). The literature search was performed using MEDLINE, EMBASE and internet key word search.

**Results:**

Of the 53 data sources identified between May and August 2016, 16 were still ongoing. Current medications (81%) and comorbidities (79%) were frequently collected, in comparison to medical imaging (36%), genetic information (30%), caregiver burden (11%) and healthcare costs (2%). Many different measurements (*n* = 108) were performed and an interesting variability among used measurements was revealed.

**Conclusions:**

Many longitudinal real world data sources on PD exist. Different types of measurements have been performed over time. To allow comparison and pooling of these multiple data sources, it will be essential to harmonize practices in terms of types of measurements.

## Background

Parkinson’s disease (PD) is a progressive neurodegenerative disease affecting approximately 630,000 people in the USA and for which no disease-modifying therapy is currently available. With the ever growing ageing population, this number is projected to almost double to 1.1 million by 2030 [1].

The Food and Drug Administration (FDA) defines “real world data” as “all data collected from sources outside of traditional clinical trials” and “real world evidence” as “all evidence derived from aggregation and analysis of real world data” [2]. Such real world evidence reflecting disease progression, treatments and outcomes under conditions of routine clinical practice is a very important resource. It can take a pivotal role to improve the understanding of the underlying disease process [3], optimize currently available therapies and develop new treatment strategies [2, 4].

Although the burden of PD and the interest of real world data are well-known [5, 6], there has not been a literature review to present the overview of longitudinal, real world studies conducted in the USA on PD patients.

There is a need for a comprehensive review to create an integrated view and assist investigators and clinicians to optimize the measurements that best match with their objectives and the already existing data sources [4, 7]. Such an assessment can be very helpful, to support a future effort to harmonize real world data collection and use the available resources in an optimal way.

The objective of this comprehensive literature review is to systematically identify and describe the longitudinal, real world data sources in PD, and to provide a summary of the key characteristics and the measurements assessed in real world studies, as a part of an effort to mobilize a harmonization process, similar to the one that already takes place in Europe.

## Methods

### Search strategy and literature sources

The search was performed on ProQuest. It was based in MEDLINE on Pubmed, in EMBASE and internet key word search between May and August 2016. Related MeSH, EMTREE and key terms were combined. Articles from peer-reviewed journals, conference abstracts and reviews were screened (AT). The search equation terms are detailed in Appendix 1.

### Study screening and selection

**Fig. 1:**
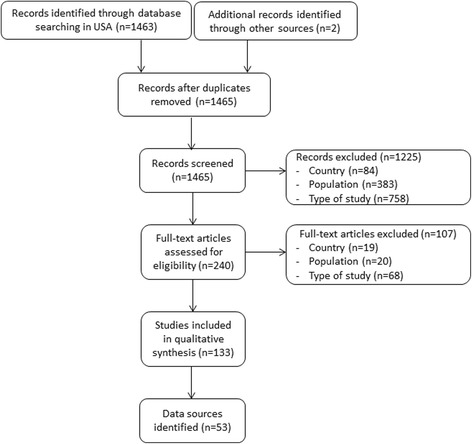
Flowchart

We included all studies including patients with a diagnosis of PD based on real world data. We restricted inclusion to only longitudinal, observational cohort studies and registries. The setting was restricted to the USA and the timing of publication in the last 10 years (2006-2016). Cohorts or registries without any publication in the last 10 years were considered as outdated. Exclusion criteria were based on population characteristics: Other diagnosis (e.g. Wolff-Parkinson-White disease or only Parkinsonian syndromes), autopsy data, and studies not focused on patients (e.g. focused on physicians). Moreover, studies without American patients or non-longitudinal studies, such as case-control, were also excluded. Only one main exclusion criterion was reported in the flow chart per excluded study (Fig. 1). No limits were applied for language.

### Data extraction

In a first step, when a publication allowed the identification of a data source of interest, the detailed information available in the publication was extracted. Information on design and setting, funding, population selection, follow-up and measurements were recorded. This was supplemented and updated via information found with an internet search of the study website, registration sites such as clinicaltrials.gov and investigators / funders’ websites. The list of all information captured is available in Appendix 2.

In a second step, a classification of measurements was performed for the following dimensions: motor and neurological function, cognition, psychiatric symptoms, activities of daily living, sleep quality, quality of life, autonomic symptoms and other. The “other” dimension gathers some known PD symptoms such as olfaction [8] not included in the previous main dimensions and more general information such as caregivers’ burden measurements. Some dimensions were subdivided in sub dimensions due to their complexity and variety (e.g. Motor and neurological symptoms is sub divided into 4 sub dimensions: global, gait and balance, fine movement and other). This classification was based on the literature [4] with one adaptation: as very few sensory markers were identified, they were gathered in the “other” category.

### Data analysis

Data source characteristics were described globally. To address the variability of sources, the description was also performed according to four main characteristics: the completion status (ongoing vs completed); the study population (Parkinson specific data sources vs “generic” data sources including both Parkinsonian patients and patients of other diagnostics); the categories of studies (investigate for motor symptoms, non-motor symptoms, biomarkers, genetics or mixed); and the country (US only vs international sources). Descriptive statistics were reported as absolute frequency and percentages.

## Results

Of 1463 records screened, 84% were excluded based on title and abstract, and 7% after review of the full-text (Fig. 1). The most frequent exclusion criterion was that studies were not longitudinal. Only 133 (9%) were included in the qualitative analysis. Of these 133 studies, data from 53 different data sources were extracted [9–61]. Only one registry was included with 52 cohorts.

### Longitudinal real world sources (Table 1)

Forty-two sources (79%) were only in the USA. Three of the 11 international sources were only in North America while the other eight included patients in the USA and Europe, and two also included Asia. Most of the sources included less than 500 PD patients (79%) for more than 5 years (51%). Although most of the sources included information about current medications (81%) and comorbidities (79%); only few collected information on medical imaging (36%), genetics (30%), caregiver’ burden (11%) and healthcare costs (2%).

**Table 1:** Overview of data sources characteristics (*n* = 53)

Characteristics		Included	Status		Country		Study population	
		All (*n* = 53)	Ongoing (*n* = 16)	Completed (*n* = 37)	USA (*n* = 42)	International (*n* = 11)	Parkinson cohort (*n* = 25)	“Generic” cohort (*n* = 28)
Size (number of Parkinsonian patients)								
	0-500	42 (79)	11 (69)	31 (84)	37 (88)	5 (45)	22 (88)	20 (71)
	500-1000	7 (13)	4 (25)	3 (8)	3 (7)	4 (36)	3 (12)	4 (14)
	>1000	4 (8)	1 (6)	3 (8)	2 (5)	2 (18)	0 (0)	4 (14)
Duration of follow-up (years)								
	<2	6 (11)	0 (0)	6 (16)	4 (10)	2 (18)	4 (16)	2 (7)
	2-5	20 (38)	4 (25)	16 (43)	16 (38)	4 (36)	13 (52)	7 (25)
	≥5	27 (51)	12 (75)	15 (41)	22 (52)	5 (45)	8 (32)	19 (68)
Dimensions assessed								
	Motor and neurological	46 (87)	12 (75)	34 (92)	36 (86)	10 (91)	25 (100)	21 (75)
	Cognition	41 (77)	13 (81)	28 (76)	36 (86)	5 (45)	17 (68)	24 (86)
	Psychiatric symptoms	38 (72)	10 (63)	28 (76)	30 (71)	8 (73)	19 (76)	17 (61)
	Activities of daily living	22 (42)	6 (38)	16 (43)	15 (36)	7 (64)	12 (48)	10 (36)
	Sleep quality	11 (21)	4 (25)	7 (19)	5 (12)	6 (55)	2 (8)	9 (32)
	Quality of life	9 (17)	4 (25)	5 (14)	5 (12)	4 (36)	6 (24)	3 (11)
	Autonomic symptoms	7 (13)	4 (25)	3 (8)	3 (7)	4 (36)	0 (0)	7 (25)
	Other	20 (38)	9 (56)	11 (30)	13 (31)	7 (64)	8 (32)	12 (43)
Other assessments								
	Current medications	43 (81)	13 (81)	30 (81)	32 (76)	11 (100)	22 (88)	21 (75)
	Comorbidities	42 (79)	14 (88)	28 (76)	31 (74)	11 (100)	20 (80)	22 (79)
	Medical imaging	19 (36)	6 (40)	13 (34)	11 (26)	8 (73)	6 (24)	13 (46)
	Genetics	16 (30)	6 (38)	10 (27)	10 (24)	6 (55)	3 (12)	13 (46)
	Caregiver burden	6 (11)	4 (27)	2 (5)	5 (12)	1 (9)	4 (16)	2 (7)
	Healthcare costs	1 (2)	1 (7)	0 (0)	0 (0)	1 (9)	1 (4)	0 (0)

Data are shown as absolute frequency (percentage)

Among the 53 sources, 16 (30%) are still ongoing. There has been an increased availability of genetic information (38% vs 27%) and caregivers’ burden data (27% vs 5%) in ongoing versus completed sources, respectively. Moreover, there has been a trend toward larger inclusions and longer durations: comparing ongoing versus completed sources, 31% vs 16% included more than 500 patients and 75% vs 41% have a duration of more than 5 years.

Likewise, US sources were smaller and shorter than international sources (88% vs 45% included less than 500 PD patients, and 52% vs 45% have a duration of more than 5 years). US sources reported more caregiver burden data than international sources (12% vs 9%) but less frequently the other assessments such as medical imaging (26% vs 73%) or genetic information (24% vs 55%).

Sources including only Parkinsonian patients were smaller (12% vs 28% included more than 500 patients) and shorter (32% vs 68% had a duration of more than 5 years) than the “generic” cohorts. Medical imaging (24% vs 46%) and genetics (12% vs 46%) were less assessed in Parkinson’s specific than in “generic” cohorts.

The 53 data sources have different objectives. Mainly the sources investigated as their primary objective: non-motor symptoms (32%), then biomarkers (21%), motor symptoms (15%) and genetics (4%). Fifteen sources (28%) investigated several of these points as first objective. The sources investigating the biomarkers as primary objective were large and recent with four sources still ongoing and four sources begun in the last 5 years. In contrast, the sources investigating the motor symptoms as primary objective were small, all with less than 500 patients and with very frequent assessment, on average twice a year.

### Measurements in real world studies in PD

The name of each included data source with its main characteristics (Table 2) and its measurements (Table 3) are presented individually. A large number of measurements (*n* = 108) was identified through this literature review and each of the 53 sources had its own unique range of measurements (Table 4). Most of the measurements were cited only once or twice. The distribution of the number of measurements over the different dimensions was not equal with only 3 different to assess autonomic symptoms and 43 to assess cognition.

**Table 2:** Overview of data sources characteristics listed in alphabetic order (*n* = 53)

Nb	Study	Acronym	Individuals included	Follow-up duration (y)	Planned follow-up	Main inclusion criteria
1	A Longitudinal Observational Follow-up of the PRECEPT Study Cohort^a^	PostCEPT	537	4		Post-RCT; under dopaminergic therapy
2	Abnormalities in metabolic network activity precede the onset of motor symptoms in Parkinson’s disease		15	4	Every 2 years	Hemi parkinsonism
3	Amyloid is linked to cognitive decline in patients with Parkinson disease without dementia		46	5	Annually	
4	Arizona Study of Aging and Neurodegenerative Disease	AZSAND	3000	ongoing		
5	Ashkenazi Jewish LRRK2 consortium cohort	LRRK2	2611	1.5	Every 12-18 months	Ashkenazi Jewish
6	Baltimore Longitudinal Study of Aging	BLSA	10,000?	ongoing	Every few years for life	Healthy
7	Boston university medical center - University of Alabama Birmingham - Washington University in Saint Louis School of medicine		80	2		>40 years
8	Central Control of Mobility in Aging	CCMA	439	ongoing	Annually	Elderly (>65 years); non demented
9	Cerebral glucose metabolic features of Parkinson disease and incident dementia: longitudinal study		50	4	Annually	Levodopa treatment
10	Charting the progression of disability in Parkinson disease		171	2	Every 6 months	>40 years; mild to moderate Parkinson’s disease
11	Clinical course in Parkinson’s disease with elevated homocysteine		97	2	Every 2 years	35-90 years without brain surgery or neurologic/psychiatric comorbidity
12	Clinical Research in Neurology (CRIN) - Emory center	CRIN	3581	15		
13	Comparative utility of the BESTest; mini-BESTest; and brief-BESTest for predicting falls in individuals with Parkinson disease: a cohort study	BESTest	80	1	Every 6 months	Without neuropsychiatric comorbidities
14	Comparison of the Agonist Pramipexole With Levodopa on Motor Complications of Parkinson’s Disease^a^	CALM-PD follow-up	301	2	Annually	Post-RCT; under dopaminergic therapy; diagnostic < 7 years
15	Contursi kindred	CONTURSI	210	?		
16	Deprenyl and Tocopherol Antioxidative Therapy of Parkinsonism^a^	DATATOP	403	6	Every 3 months	Early phase; postRCT; 30-79 years
17	Depression in Parkinson’s disease		685	3.9	Annually	
18	Dopamine agonist withdrawal syndrome in Parkinson disease^a^	DAWS	93	0.25	Annually	Non demented
19	Einstein Aging Study (Bronx Aging Study)	EAS	791	ongoing	Every 12 to 18 months	Elderly (>70 years)
20	Emergence and evolution of social self-management of Parkinson’s disease		120	3	Every 6 months	Non demented
21	Hallucinations and sleep disorders in PD: ten-year prospective longitudinal study		89	10	0; 6 months; 18 months; 4 years; 6 years; 10 years	24-h caregiver; without neuroleptic treatment; without some comorbidities
22	Harvard Alumni Health Study		500,002	77	1962; 1966; 1972; 1988; 1993	Harvard students
23	Health Professionals Follow-up Study	HPFS	51,529	ongoing	Biannually	Men; healthy; 40-75 years
24	Honolulu Asia Aging Study	HAAS	3741	15	3 times between 1994 and 2001	Elderly Japanese-American men
25	Longitudinal study of normal cognition in Parkinson disease		141	6	Biannual for 4 years and annual after	Normal cognition at baseline
26	Long-term outcomes of bilateral subthalamic nucleus stimulation in patients with advanced Parkinson’s disease^a^		33	2	0 –3 –6 –12 –18 – 24 months	Advanced phase with deep brain stimulation
27	Loss of ability to work and ability to live independently in Parkinson’s disease		495	10		
28	Major life events and development of major depression in Parkinson’s disease patients	PEG study	221	4	Annually	New onset (within 3 years)
29	Mayo Clinic cohort study of Personality and Aging (including Rochester Epidemiology project)		7216	29.2	Historically for life	20-69 years
30	Mayo clinic study of aging (Olmsted county resident) - Rochester Epidemiology project indexing system	MCSA	2739	ongoing		
31	Molecular Epidemiology of Parkinson’s Disease	MEPD	1600	ongoing		>40 years
32	Mood and motor trajectories in Parkinson’s disease: multivariate latent growth curve modeling		186	1.5	6 months; 18 months	
33	Mood and Subthalamic Nucleus Deep Brain Stimulation^a^	MOST	91	1		Deep brain stimulation eligible; not demented
34	Morris K Udall Parkinson’s Disease Research Center of Excellence cohort - Veteran affair	Udall	314	ongoing		Elderly (>60 years)
35	National Parkinson Foundation Quality Improvement Initiative	NPF-QII	10,000	on going		
36	NeuroGenetics Research Consortium	NGRC	3072	>10		
37	Nurses’ Health Study	NHS	280,000	ongoing	Every 2 years	Women; healthy; 19-51 years
38	Oxford Parkinson’s Disease Centre	OPDC	1500	1.5	18 months	
39	Parkinson’s Associated Risk Study	PARS	10,000	ongoing		Elderly (>60 years)
40	Parkinson’s Disease Biomarkers Program	PDBP	1436	ongoing		Evidence of response to dopaminergic medication
41	Parkinson’s Disease Research Education and Clinical Center - Parkinson’s Genetic Research Study	PADRECCS - PaGeR	1880	ongoing		
42	Parkinson’s disease: increased motor network activity in the absence of movement	NMRP	12	4.4	Every 2 years	Non demented; tremor-dominant clinical manifestations; without some comorbidities
43	Parkinson’s Progression bioMarkers Initiative	PPMI	748	ongoing	Every 3 months the first year then every 6 months	Untreated recently diagnosed
44	Prospective cohort study of impulse control disorders in Parkinson’s disease	ICD-PD	164	4		Non demented
45	Rate of 6-18Ffluorodopa uptake decline in striatal subregions in Parkinson’s disease		37	4	Every 1 to 2 years	
46	Religious Order Study	ROS	>1100	>7	Annually	Elderly; religious clergy
47	Rush Memory and Aging Project	RMAP	1556	5	Annually	Elderly without know dementia
48	Study of Osteoporotic Fractures (SOF) Research Group	SOF	9704	>6	Tri-annually	Women; Elderly (>65 years)
49	The effect of age of onset of PD on risk of dementia		440	4	Annually	Elderly (>65 years)
50	University of California Los Angeles Center for Genes and Environmental in Parkinson’s Disease	UCLA CGEP	363	5		Diagnostic >3 years
51	University of Miami Brain Endowment Bank	UM/BEB	150	ongoing	Annually	Consent to donate brain
52	UPDRS activity of daily living score as a marker of Parkinson’s disease progression		162	6	Every 2 years	
53	Washington Heights-Inwood Columbia Aging	WHICAP	2776	3.7	Annually	Elderly (>65 years)

Post-RCT = Open label extension after a Randomized Controlled Trial

^a^Treatment directed data sources

**Table 3:** Overview of data source measurements and of the number of evaluations or assessments applied (*n* = 53)

Nb	Study	Motor and neurological	Cognition	Psychiatry	Activities of daily living	Sleep	Quality of life	Autonomic	Other
1	A Longitudinal Observational Follow-up of the PRECEPT Study Cohort	3	4	3	1	0	0	0	0
2	Abnormalities in metabolic network activity precede the onset of motor symptoms in Parkinson’s disease	2	0	0	0	0	0	0	0
3	Amyloid is linked to cognitive decline in patients with Parkinson disease without dementia	2	14	1	0	0	0	0	0
4	Arizona Study of Aging and Neurodegenerative Disease	4	12	3	0	1	0	1	1
5	Ashkenazi Jewish LRRK2 consortium cohort	3	2	2	2	1	0	1	1
6	Baltimore Longitudinal Study of Aging	0	2	3	0	0	0	0	0
7	Boston university medical center - University of Alabama Birmingham - Washington University in Saint Louis School of medicine	9	1	1	0	0	1	0	0
8	Central Control of Mobility in Aging	2	1	1	0	0	0	0	0
9	Cerebral glucose metabolic features of Parkinson disease and incident dementia: longitudinal study	1	6	0	0	0	0	0	0
10	Charting the progression of disability in parkinson disease	9	1	1	0	0	1	0	0
11	Clinical course in Parkinson’s disease with elevated homocysteine	1	9	1	1	0	0	0	0
12	Clinical Research in Neurology (CRIN) - Emory center	0	1	0	0	0	0	0	0
13	Comparative utility of the BESTest; mini-BESTest; and brief-BESTest for predicting falls in individuals with Parkinson disease: a cohort study	5	0	0	0	0	0	0	0
14	Comparison of the Agonist Pramipexole With Levodopa on Motor Complications of Parkinson’s Disease	3	1	2	2	1	3	0	0
15	Contursi kindred	1	1	1	1	1	0	1	1
16	Deprenyl and Tocopherol Antioxidative Therapy of Parkinsonism	2	5	0	0	0	0	0	0
17	Depression in Parkinson’s disease	2	0	1	1	0	0	0	0
18	Dopamine agonist withdrawal syndrome in parkinson disease	2	1	4	1	0	1	0	0
19	Einstein Aging Study (Bronx Aging Study)	2	11	1	0	0	0	0	0
20	Emergence and evolution of social self-management of Parkinson’s disease	2	2	1	1	0	4	0	0
21	Hallucinations and sleep disorders in PD: ten-year prospective longitudinal study	2	1	1	0	1	0	0	0
22	Harvard Alumni Health Study	0	0	0	0	0	0	0	0
23	Health Professionals Follow-up Study	0	0	0	0	0	0	0	0
24	Honolulu Asia Aging Study	2	4	2	0	1	0	1	1
25	Longitudinal study of normal cognition in Parkinson disease	2	6	2	1	0	0	0	0
26	Long-term outcomes of bilateral subthalamic nucleus stimulation in patients with advanced Parkinson’s disease	2	2	2	2	0	0	0	0
27	Loss of ability to work and ability to live independently in Parkinson’s disease	2	0	1	1	0	0	0	0
28	Major life events and development of major depression in Parkinson’s disease patients	1	2	2	0	0	0	0	0
29	Mayo Clinic cohort study of Personality and Aging (including Rochester Epidemiology project)	0	0	4	0	0	0	0	0
30	Mayo clinic study of aging (Olmsted county resident) - Rochester Epidemiology project indexing system	1	10	3	0	1	0	1	1
31	Molecular Epidemiology of Parkinson’s Disease	1	3	0	0	0	0	0	0
32	Mood and motor trajectories in Parkinson’s disease: multivariate latent growth curve modeling	1	0	2	0	0	0	0	0
33	Mood and Subthalamic Nucleus Deep Brain Stimulation	2	0	7	0	0	0	0	0
34	Morris K Udall Parkinson’s Disease Research Center of Excellence cohort - Veteran affair	2	3	2	1	0	1	0	1
35	National Parkinson Foundation Quality Improvement Initiative	3	2	0	0	0	1	0	1
36	NeuroGenetics Research Consortium	1	1	1	0	0	0	0	0
37	Nurses’ Health Study	0	5	0	0	0	0	0	0
38	Oxford Parkinson’s Disease Centre	6	3	2	1	2	1	0	2
39	Parkinson’s Associated Risk Study	0	0	2	0	0	0	0	1
40	Parkinson’s Disease Biomarkers Program	4	3	6	1	6	5	1	3
41	Parkinson’s Disease Research Education and Clinical Center - Parkinson’s Genetic Research Study	3	1	0	1	0	0	0	0
42	Parkinson’s disease: increased motor network activity in the absence of movement	2	1	0	0	0	0	0	0
43	Parkinson’s progression biomarkers initiative	1	5	4	2	2	0	1	2
44	Prospective cohort study of impulse control disorders in Parkinson’s disease	2	1	2	1	0	0	0	0
45	Rate of 6-18Ffluorodopa uptake decline in striatal subregions in Parkinson’s disease	2	1	0	0	0	0	0	0
46	Religious Order Study	6	11	4	1	0	0	0	0
47	Rush Memory and Aging Project	5	1	3	1	1	0	0	2
48	Study of Osteoporotic Fractures (SOF) Research Group	2	1	1	0	0	0	0	2
49	The effect of age of onset of PD on risk of dementia	1	6	1	0	0	0	0	0
50	University of California Los Angeles Center for Genes and Environmental in Parkinson’s Disease	2	1	1	0	0	0	0	0
51	University of Miami Brain Endowment Bank	1	0	0	1	0	0	0	2
52	UPDRS activity of daily living score as a marker of Parkinson’s disease progression	1	0	1	1	0	0	0	0
53	Washington Heights-Inwood Columbia Aging	1	6	0	1	0	0	0	0

**Table 4:** Measurements classification and use in data sources (*n* = 108)

Dimension	Measurement acronym	Measurement full name	Data sources (number and numbering)
Motor and neurological (*n* = 46)			
Global	H&Y	Hoehn and Yahr	(*n* = 30) °1,2,3,4,5,7,9,10,13,14,16,17,18,20,21,25,26,27,31,33,34,35,38,40,41,42,44,45,50,51
	UPDRS-III	Unified Parkinson’s Disease Rating Scale - motor examination	(*n* = 41) °1,2,3,4,5,7,8,10,11,13,14,16,17,18,19,20,21,24,25,26,27,28,30,32,33,34,35,36,38,40,41,42,43,44,45,46,47,49,50,52,53
	UPDRS-IV	Unified Parkinson’s Disease Rating Scale - motor complications	(*n* = 2) n°1,14
Gait and balance		Berg balance test	(*n* = 2) n°7,10
		Flamingo test	(*n* = 1) n°38
	FGA	Functional Gait Assessment	(*n* = 2) n°7,10
	FOGQ	Freezing of gait questionnaire	(*n* = 2) n°7,10
		Gait speed	(*n* = 4) n°7,8,10,46
	PIGD	Postural Instability / Gait Difficulty scale	(*n* = 2) n°5,40
		Tandem gait	(*n* = 1) n°48
	TUG	Time Up and Go test	(*n* = 6) n°7,10,35,38,40,47
		Walk test	(*n* = 5) n°7,10,46,47,48
Fine movement		Finger tapping	(*n* = 3) n°4,46,47
		Purdue pegboard test	(*n* = 6) n°4,7,10,38,46,47
		Reaction time	(*n* = 1) n°24
		Unknown	(*n* = 1) n°15
Cognition (*n* = 41)			
Global	ACE	Addenbrooke’s Cognitive Examination	(*n* = 1) n°40
	AD-8	Ascertian Dementia 8-item Informant	(*n* = 1) n°31
	BDRS	Blessed Dementia Rating Scale	(*n* = 2) n°19,53
	CAMCOG	Cambridge Cognitive Assessment	(*n* = 1) n°49
	CASI	Cognitive Abilities Screening Instrument	(*n* = 1) n°24
	CDR	Clinical Dementia Rating scale	(*n* = 5) n°3,4,6,19,30,53
		Clock drawing test	(*n* = 1) n°4
	DRS2	Dementia Rating Scale 2	(*n* = 6) n°4,19,25,26,34,53
	HDS	Hasegawa Dementia Rating Scale	(*n* = 1) n°24
	MDRS	Mattis Dementia Rating Scale	(*n* = 2) n°4,26
	MMSE	Mini Mental State Examination	(*n* = 30) °1,3,4,5,7,9,10,11,12,14,15,16,18,20,21,24,26,28,31,34,36,37,38,42,44,45,46,47,48,50
	MoCA	Montreal Cognitive Assessment	(*n* = 9) n°1,4,5,20,34,38,40,41,43
	IQCODE	Informant Questionnaire on Cognitive Decline in Elderly	(*n* = 1) n°24
	SPMSQ	Short Portable Mental Status Questionnaire	(*n* = 1) n°40
	TICS-M	Telephone Interview Cognitive Status Modified	(*n* = 2) n°31,37
Attention/ Working memory		Digit span	(*n* = 6) n°3,4,11,30,37,46
		STROOP test	(*n* = 2) n°4,11
Executive function		Comprehension	(*n* = 2) n°28,49
	RBANS	Repeatable Battery for Assessment of Neuropsychological Status	(*n* = 1) n°8
		Symbol digit	(*n* = 3) n°16,43,46
		Trail Making Test	(*n* = 4) n°3,4,19,30
		Verbal fluency	(*n* = 12) n°3,9,11,19,25,30,35,37,38,43,46,49
Language	BNT	Boston Naming Test	(*n* = 5) n°3,25,30,37,46
	COWA	Controlled Oral Word Association	(*n* = 4) n°1,3,4,11
	FAS	Letter-Number Sequencing and Phonemic verbal fluency	(*n* = 2) n°11,25
		Naming	(*n* = 1) n°49
	NART	American National Adult Reading test	(*n* = 2) n°3,46
	WAIS	Wechlser Adult Intelligence Scale	(*n* = 6) n°3,4,9,11,19,30
Memory	BIMC	Blessed Information Memory Concentration	(*n* = 2) n°6,19
	FCSRT	Free and Cue Selective Reminding Test	(*n* = 2) n°3,19
	FOME	Fuld Object Memory Evaluation	(*n* = 1) n°19
	HVLT	Hopkins Verbal Learning test	(*n* = 3) n°11,25,43
		Memory	(*n* = 5) n°3,16,35,46,53
	RAVLT	Rey auditory verbal learning test	(*n* = 3) n°1,4,30
		Recall	(*n* = 2) n°46,49
	WMS	Wechsler Memory Scale	(*n* = 2) n°9,30
Visual-spatial	BVRT	Benton Visual Retention Test	(*n* = 1) n°9
	CPM	*Raven’s coloured progressive matrices*	(*n* = 2) n°19,46
	JLO	Benton Judgement Line Orientation	(*n* = 4) n°4,25,43,46
		Orientation	(*n* = 1) n°53
	PARR	Picture Arrangement subtest	(*n* = 1) n°9
	ROCF	Rey-Osterrieth Complex Figure test recall	(*n* = 1) n°11
		Visual attention	(*n* = 1) n°19
		Unknown	(*n* = 1) n°15
Psychiatric symptoms (*n* = 38)			
Depression / Anxiety	AS	Apathy Evaluation Scale	(*n* = 3) n°4,32,33
	BAI	Beck Anxiety Inventory	(*n* = 4) n°18,30,33,44
	BDI	Beck Depression Inventory	(*n* = 9) n°5,11,18,26,30,32,33,36,44
	CESD-10	Center for Epidemiological Studies Depression Scale	(*n* = 3) n°24,39,47
	GDS	Geriatric Depression Screening scale	(*n* = 17) n°1,3,4,5,7,8,10,14,20,25,26,28,34,40,43,48,50
	HAM-A	Hamilton Anxiety Rating Scale	(*n* = 2) n°33,40
	HDRS	Hamilton Depression Rating Scale	(*n* = 3) n°4,15,33
	Leeds	Leeds anxiety and depression scale	(*n* = 1) n°38
	SCID	Structured Clinical Interview - Depression	(*n* = 2) n°28,40
	STAI	State Trait Anxiety Inventory	(*n* = 4) n°18,24,39,43
	UPDRS-I	Unified Parkinson’s Disease Rating Scale - mentation behavior and mood	(*n* = 7) n°1,14,17,25,27,43,52
	ZUNG	Zung depression scale	(*n* = 1) n°19
TOC	OCI-R	Obsessive-Compulsive Inventory – Revised	(*n* = 1) n°18
	QUIP	Questionnaire for impulsive-compulsive disorders in parkinson’s disease-rating scale	(*n* = 2) n°40,43
	YBOCS	Yale-Brown obsessive-compulsive scale	(*n* = 1) n°33
Other	CoNeg	composite negative score	(*n* = 1) n°29
	MMPI	Multiphasic Personality Inventory	(*n* = 1) n°29
	NPI	NeuroPsychiatric Inventory questionnaire	(*n* = 3) n°1,34,47
	QABB	Questionnaire About Buying Behaviour	(*n* = 1) n°40
	Rush	Rush Hallucination Inventory	(*n* = 1) n°21
	SCS	Sexual Compulsivity Scale	(*n* = 1) n°40
	YMRS	Young Mania Rating Scale	(*n* = 1) n°33
		Unknown	(*n* = 4) n°6,15,46,49
Activities of daily living (*n* = 22)			
	ACS	Activity Card Sort	(*n* = 1) n°20
	ADCS-ADL	Alzheimer’s Disease Cooperative Study ADL Inventory	(*n* = 1) n°25
	IADL	Katz Instrumental Activity of Daily Living	(*n* = 2) n°46,47
	S&E	Schwab & England activities of daily living scale	(*n* = 10) n°5,14,18,26,34,38,41,43,44,53
	UPDRS-II	Unified Parkinson’s Disease Rating Scale - self-evaluation of the activities of daily living	(*n* = 9) n°1,5,11,14,26,27,40,43,52
		Unknown	(*n* = 3) n°15,17,51
Sleep quality (*n* = 11)			
		Actigraphy	(*n* = 1) n°47
	ESS	Epworth Sleepiness Scale	(*n* = 4) n°5,14,38,43
	FSS	Fatigue Severity Scale	(*n* = 1) n°40
	ISI	Insomnia Severity Index	(*n* = 1) n°40
	MSQ	Mayo clinic Sleep Questionnaire	(*n* = 2) n°4,30
	PDSS	Parkinson’s disease sleep scale	(*n* = 1) n°40
	PSQI	Pittsburg Sleep Quality Index	(*n* = 2) n°21,40
	RBDSQ	REM Sleep Behaviour Disorder Screening Questionnaire	(*n* = 2) n°38,43
	SA-SDQ	Sleep Apnea Scale of Sleep Disorders Questionnaire	(*n* = 1) n°40
	SSS	Stanford Sleepiness Scale	(*n* = 1) n°40
		Unknown	(*n* = 2) n°15,24
Quality of life (n = 9)			
	EQ-5D	Euro Quality of Life 5 Dimension questionnaire	(*n* = 2) n°14,38
	Neuro-QOL	Quality of Life in Neurological Disorders	(*n* = 1) n°34
	NHP	Nottingham Health Profile	(*n* = 1) n°20
	PDQUALIF	Parkinson’s Disease Quality of Life Scale	(*n* = 3) n°14,18,40
	PDQ-39	39-item Parkinson’s disease quality of life	(*n* = 5) n°7,10,20,35,40
	PIMS	Parkinson’s Impact Scale	(*n* = 1) n°40
	SF-12	The 12 item Short Form health survey	(*n* = 2) n°14,20
	SF-36	The 36 item Short Form health survey	(*n* = 1) n°40
	SWAL-QOL	Swallow-specific quality of life	(*n* = 1) n°40
Autonomic symptoms (n = 7)			
		Bowel movement	(*n* = 1) n°24
	COMPASS	Composite autonomic symptom Scale	(*n* = 1) n°40
	SCOPA-AUT	Scales for outcomes of Parkinson’s Disease – autonomic symptoms	(*n* = 3) n°4,5,43
		Unknown	(*n* = 2) n°15,30
Other (*n* = 20)			
Olfaction	Brief-SIT	Brief Smell Identification Test	(*n* = 2) n°24,47
		16-item sniffin’ Sticks Odour Identification test	(*n* = 1) n°38
	UPSIT	University of Pennsylvania Smell Identification Test	(*n* = 6) n°1,4,5,34,39,43
Restless legs syndrome	CH-RLSQ	Cambridge-Hopkins Restless Legs Syndrome Diagnostic Questionnaire	(*n* = 1) n°40
	IRLSSG	Instrument for the Assessment of Restless Legs Syndrome Severity	(*n* = 1) n°4
Caregiver	CSI	caregiver strain index	(*n* = 1) n°35
		deJong-Gierveld Loneliness Scale	(*n* = 1) n°47
	MCSI	Multidimensional Caregiver Strain Index	(*n* = 1) n°35
		Caregiver interview	(*n* = 1) n°21
Other		Agonal state questionnaire	(*n* = 1) n°51
	CGI	Clinical Global Impression scale	(*n* = 1) n°38
	CIRS	Chronic Illness Resource Survey	(*n* = 1) n°20
	GHS	Global Health Score	(*n* = 1) n°8
	GIS	Global Impression Scale	(*n* = 1) n°51
		Howard-Dohlman device	(*n* = 1) n°48
	MNA	*Mini Nutritional Assessment*	(*n* = 1) n°40
	MOS	*Medical outcome study*	(*n* = 1) n°20
	MSSSS	Medical Outcomes Study Social Support Scale	(*n* = 1) n°28
		Pain	(*n* = 1) n°40
	PASE	Physical Activity Scale for the Elderly	(*n* = 3) n°7,10,43
	SRRS	Social Readjustment Rating scale	(*n* = 1) n°28
	SSCI	Stigma Scale for Chronic Illness	(*n* = 1) n°20
		Tremor rating	(*n* = 1) n°4
		Visual acuity	(*n* = 1) n°48
		Unknown	(*n* = 1) n°15

Most sources assessed motor and neurological functions (87%), cognition (77%) and psychiatric symptoms (72%). Activity level (42%), sleep quality (21%), quality of life (17%) and autonomic symptoms (13%) were reported to a lesser extent. The most commonly measurements used to assess motor and neurological symptoms were the Unified Parkinson’s Disease Rating Scale part III (UPDRS-III, 77% of included data sources) and the Hoehn and Yahr scale (H&Y, 57% of included data sources)(Table 4). To evaluate the cognitive impairment, the Mini Mental State Examination (MMSE, 57%) was the most frequent. Those most frequently used to assess psychiatric symptoms were the Geriatric Depression Scale (GDS, 32%) and Beck Depression Inventory (BDI, 15%). For the other dimensions, the most commonly used measurements were: the Epworth Sleepiness Scale (ESS, 8%, for sleep), the Schwab and England (S&E, 19%, for activities of daily living), the 39-item Parkinson’s disease Quality of life (PDQ-39, 9%, for the quality of life) and the autonomic part of the Scales for outcomes of Parkinson’s disease (SCOPA-AUT, 6%, for autonomic symptoms). In absolute frequency, the use of ESS, PDQ-39 and SCOPA-AUT is very low, even if they were the most frequently used measurements in their dimension.

The analysis reveals some interesting differences between sources on the number of measurements applied by dimension. Some sources evaluate only one dimension (source n°13) when others evaluate seven dimensions (source n°43). Completed sources have more frequent measurements of motor and neurological symptoms (92% vs 75%), psychiatric symptoms (76% vs 63%) and activities of daily living (43% vs 38%) than ongoing sources. US sources evaluate more frequently the cognitive impairment then international sources (86% vs 45%) but less frequently all the other dimensions. “Generic” sources evaluate three dimensions more frequently than specific sources including only Parkinsonian patients: cognition (86% vs 68%), sleep (32% vs 8%) and autonomic symptoms (25% vs 0%).

Lastly, the frequencies of these assessments are dependent on the primary objective of the sources but with an important overlap: 100% of the sources investigating motor symptoms used measurements of motor symptoms and mainly the UPDRS-III, but they also frequently assessed cognition (88%), sleep (25%) and quality of life (25%). The sources investigating non-motor symptoms frequently assessed cognition (82%), psychiatric symptoms (88%) most of the time with, respectively, the GDS (41%) and the MMSE (65%). The two genetic sources have several patient reported outcomes and they both measured motor and psychiatric symptoms.

Some measurements were used more often for some above-mentioned objectives. While the GDS and the UPDRS-III were used specifically in sources investigating, respectively, the non-motor symptoms and the motor symptoms as a primary objective, the BDI and the H&Y were used in sources investigating the other objectives.

## Discussion

A large number of longitudinal real world data sources for PD have been identified. There is no consistency of the dimensions assessed, nor of the measurements used across sources, reflecting the absence of harmonization on the optimal choice of measurements.

There are a number of issues with collecting real world data such as limited size of the databases [1], inability to accurately determine specific outcomes [62], and more chance of bias and confounding factors [5]. Nevertheless, they have an important role to play in the evaluation of epidemiology, burden of disease and treatments patterns [6]; and in assisting health-care decision-makers, especially related to coverage and payment decisions [63]. In this context, a harmonization seems necessary. These results are quite consistent with those observed in Europe where a “consensus on domains incorporated in different studies [was observed] with a substantial variability in the choice of the evaluation method” [4]. There are a number of possible explanations for this absence of harmonization and some of them are discussed here.

First of all, some dimensions are broad. In consequence many measurements are available according to each source objective, design and population. This heterogeneity probably reflects both the absence of harmonization and the complexity of the evaluation of a dimension like cognition [64]. A single measurement cannot assess all necessary information. For example, the combination of patient reported outcomes and medical reported outcomes can be very informative and complement one another. In a consistent manner, the combination of Parkinson specific and generic measurements can be a necessity especially for “generic” data sources including not only Parkinsonian patients. In another example, while the objectives of the UPDRS-III and the H&Y (or of the GDS and the BDI) are close, the difference of their use according to the study primary objective of the source seems more linked to the investigator choice than to the suitability of the measurement.

Secondly, PD is characterized by several initial system disorders and treatment complications [65]. To date, motor subtyping has dominated the landscape of PD research but non-motor dimensions evaluations are increasing [9, 66], and thus the number of dimensions to evaluate. For non-motor dimensions, some have validated measurements such as psychiatry [67], activity disability [7], sleep [68] or quality of life [69]; but others have no clear review of validated and used scales [4]. Among the psychiatric scales, the two most frequently used were the GDS and the BDI. This finding highlights the well-known relationship between PD and depression, and the fact that when validated scales [70] are available, a harmonization of practice is observed. The lack of evaluation and validation of the measurements in PD is probably partly a source of such an heterogeneity.

Thirdly, clinical research purposes and outcomes are in permanent evolution over time [71, 72], as highlighted by the many differences between completed and ongoing sources. New trends are not well covered right now, either due to lack of measurements or due to lack of capture (i.e. utilization of available measurements in databases). Among the most important of those are the genetic testing, the caregiver burden and the costs. The important development of genetic testing has come in the last few years, with an increase of the mutations and treatment discoveries such as LRRK2 and its kinase inhibitors. But research is necessary to understand the role of genetic mutations in PD [73]. Sources based on caregiver burden and relevant validated measurements are very limited [7]. But the interest for these data is growing with the recognition of their physical, emotional and economic burden [74]. The only data source identified as measuring healthcare costs associated with PD was ongoing. It probably reflects both the recent growing interest of health economic evaluation and the fact that this type of study is more often conducted in automated healthcare databases [75].

Fourthly, there is a possible improvement of the access to the data source details. Given information is fragmented between different sources of information and study protocols or outcomes lists are not always available. In consequence identifying and gathering this information to produce an integrated view can be really difficult.

Finally, the variability of our results is greater than in the European study. This may be because the classification is based on dimensions assessing mostly symptoms, 5 out of 8 dimensions. This classification probably more appropriate for data sources with a primary objective of treatment evaluation (e.g. open-label extension), which are a minority of the included sources. The classification may not be as applicable to assess other data sources focused on the evaluation of burden. Real world evidence collection is done for various purposes and such a restricted classification can lead to ambiguous conclusions. It can lead to a perception of consensus while actually missing important aspects such as burden, function or complications of treatments.

Our study has several limitations. First of all, only one reader has conducted the record selection and the data extraction unlike systematic reviews. Nevertheless, the search methods identified a large number of PD data sources for extraction and comparison. No contact was established with investigators of the included studies to confirm data extraction results. To address this issue, a second step has been performed after the data extraction from the publications, to update and complete the published information with all other available sources. At risk/prodromal cohorts have not been separated from clinical PD cohorts, but the distinction between these two subgroups has recently been described as artificial [4].

Our study has several strengths. It is the first review of existing real world longitudinal data sources on PD in USA to our knowledge. Moreover, it was performed with broad research criteria and without any limitation on language, type of publication or type of measurements. This review creates an integrated view and should assist investigators and clinicians to identify and optimize the measurements that best match with their objectives and the already existing data sources.

## Conclusion

In conclusion, many longitudinal real world data sources on PD exist. Different types of measurements have been used over time. To allow comparison and pooling of these multiple data sources, it will be essential to harmonize practices in terms of types of measurements.

## Appendix 1

Search strategy.

Equation 1: Disease selection

(EMB.EXACT(“Parkinson disease”) OR MESH.EXACT(“Parkinson Disease”) OR ab(“Parkinson*”)

OR ti(“Parkinson*”) OR EMB.EXACT(“antiparkinson agent”) OR MESH.EXACT(“Antiparkinson Agents”)) AND (human(yes) AND human(yes)).

Equation 2: Disease exclusion

(MESH.EXACT(“Parkinson Disease, Postencephalitic”) OR MESH.EXACT(“Parkinson Disease, Secondary”) OR EMB.EXACT(“Wolff Parkinson White syndrome”) OR EMB.EXACT(“experimental parkinsonism”) OR EMB.EXACT(“parkinsonism”) OR EMB.EXACT(“MPTPinduced parkinsonism”)) AND (human(yes) AND human(yes))

Equation 3: Study type selection

((EMB.EXACT(“register”) OR EMB.EXACT(“long term care”) OR EMB.EXACT(“retrospective study”) OR EMB.EXACT(“prospective study”) OR EMB.EXACT(“cohort analysis”) OR EMB.EXACT(“clinical practice”) OR EMB.EXACT(“longitudinal study”)) OR (MESH.EXACT(“Cohort Studies”) OR MESH.EXACT(“Registries”) OR MESH.EXACT(“Longitudinal Studies”) OR MESH.EXACT(“Long-Term Care”) OR MESH.EXACT (“Retrospective Studies”) OR MESH.EXACT(“Prospective Studies”) OR MESH.EXACT(“Practice Patterns, Physicians’”))) OR (((longitudinal OR retrospective OR prospective OR cohort OR “follow up” OR observational OR naturalistic OR “cross*sectional” OR epidemio* OR database) NEAR/1 (study OR studies)) OR “cohort analysis” OR “registry” OR “register*” OR “real-world” OR “treatment pattern*” OR “survey*” OR “medical records” OR “population-correlation” OR “population-based” OR “population-level”)

Equation 4: Study type exclusion

((MESH.EXACT(“Case-Control Studies”) OR MESH.EXACT(“Controlled Before-After Studies”) OR.

MESH.EXACT(“Feasibility Studies”) OR MESH.EXACT(“Clinical Trial”) OR MESH.EXACT(“Organizational Case Studies”) OR MESH.EXACT(“Evaluation Studies”)) OR (EMB.EXACT (“major clinical study”) OR EMB.EXACT(“in vivo study”) OR EMB.EXACT(“evaluation study”) OR EMB.EXACT(“in vitro study”) OR EMB.EXACT(“first in human study”) OR EMB.EXACT(“experimental study”) OR EMB.EXACT(“case study”) OR EMB.EXACT(“clinical study”) OR EMB.EXACT(“intervention study”) OR EMB.EXACT(“case control study”))).

Equation 5: Combination of the previous equation

(Equation 1 NOT Eq. 2) AND (Eq. 3 NOT Eq. 4)

Equation 6: Country selection

GI(“United States*”) OR ti(“America*”) OR ab(“America *”) OR ab(“usa”) OR ti(“usa”) OR ab(“us”) OR ti(“us”) OR ab(“u.s”) OR ti(“u.s”).

Equation 7: Application of the combination equation to the country of interest

Equation 6 AND Eq. 6

## Appendix 2

List of outcomes extracted.

Acronym

○Full name

○Country (−ies)

○Database size (total number of patients and number of Parkinsonian patients)

○Database type

○Name of investigator (corresponding author of the publication, reference person)

○Funder(s)

○Medical imaging

○Scales list

○Scales dimension 1: Activities of daily living

○Scales dimension 2: Cognition

○Scales dimension 3: Motor or neurologic symptoms

○Scales dimension 4: Psychiatric symptoms

○Scales dimension 5: Sleep quality

○Scales dimension 6: Quality of life

○Scales dimension 7: Autonomic symptoms

○Scales dimension 8: Other

○Healthcare costs

○Genetics

○Comorbidities

○Current medications

○Severity of disease

○Caregiver burden

○Date of beginning of the study

○Date of end of the study

○Duration of follow-up

○Planned follow-ups

○Particular inclusion criteria

## Abbreviations

BDI: Beck Depression Inventory
ESS: Epworth Sleepiness Scale
FDA: Food and Drug Administration
GDS: Geriatric Depression Scale
H&Y: Hoehn and Yahr scale
MMSE: Mini Mental State Examination
PD: Parkinson’s disease
PDQ-39: 39-item Parkinson’s disease Quality of life
S&E: Schwab and England
SCOPA-AUT: autonomic part of the Scales for outcomes of Parkinson’s disease
UPDRS-III: Unified Parkinson’s Disease Rating Scale part III
USA: United States of America
